# Effect modification by sex of genetic associations of vitamin C related metabolites in the Canadian Longitudinal study on aging

**DOI:** 10.3389/fgene.2024.1411931

**Published:** 2024-07-31

**Authors:** Rebecca Lelievre, Mohan Rakesh, Pirro G. Hysi, Julian Little, Ellen E. Freeman, Marie-Hélène Roy-Gagnon

**Affiliations:** ^1^ School of Epidemiology and Public Health, University of Ottawa, Ottawa, ON, Canada; ^2^ Section of Ophthalmology, School of Life Course Sciences, King’s College London, London, United Kingdom

**Keywords:** vitamin C, metabolites, GWAS, gene-environment interaction, CLSA

## Abstract

**Introduction:** Vitamin C is an essential nutrient. Sex differences in serum vitamin C concentrations have been observed but are not fully known. Investigation of levels of metabolites may help shed light on how dietary and other environmental exposures interact with molecular processes. O-methylascorbate and ascorbic acid 2-sulfate are two metabolites in the vitamin C metabolic pathway. Past research has found genetic factors that influence the levels of these two metabolites. Therefore, we investigated possible effect modification by sex of genetic variant-metabolite associations and characterized the biological function of these interactions.

**Methods:** We included individuals of European descent from the Canadian Longitudinal Study on Aging with available genetic and metabolic data (n = 9004). We used linear mixed models to tests for genome-wide associations with O-methylascorbate and ascorbic acid 2-sulfate, with and without a sex interaction. We also investigated the biological function of the important genetic variant-sex interactions found for each metabolite.

**Results:** Two genome-wide statistically significant (*p* value < 5 × 10^−8^) interaction effects and several suggestive (*p* value < 10^–5^) interaction effects were found. These suggestive interaction effects were mapped to several genes including *HSD11B2*, associated with sex hormones, and *AGRP*, associated with hunger drive. The genes mapped to O-methylascorbate were differently expressed in the testis tissues, and the genes mapped to ascorbic acid 2-sulfate were differently expressed in stomach tissues.

**Discussion:** By understanding the genetic factors that impact metabolites associated with vitamin C, we can better understand its function in disease risk and the mechanisms behind sex differences in vitamin C concentrations.

## 1 Introduction

Vitamin C consumption and its effects on aging have been extensively studied (Balboa-Castillo et al., 2018; Carr and Zawari, 2023). There has been evidence to suggest that older adults do not consume a high enough concentration, and this may lead to increased risk of frailty and other conditions due to the immune effects of the vitamin (Carr and Maggini, 2017; Carr and Zawari, 2023). Past research has also found differences between serum vitamin C concentrations in males and females, with males more likely to have lower concentrations (Schleicher et al., 2009; Carr and Lykkesfeldt, 2023). Several theories have been proposed to account for this difference, including external factors such as body size and lifestyle factors and internal or molecular factors such as sex hormones (Travica et al., 2020). Some studies which have noted the influence of sex hormones on vitamin C concentrations have relied on cellular and animal models (Nathani and Nath, 1972; Kume-Kick and Rice, 1998; Travica et al., 2020). One recent cross-sectional study in infertile men found there was an inverse association between serum ascorbic acid and luteinizing hormone levels, however, the relationship between vitamin C and sex hormones in humans needs to be further explored (Rastegar Panah et al., 2023).

The exposome is the totality of all environmental exposures in someone’s lifetime and how they influence disease (Wild, 2005). One way to quantify a portion of the exposome is through the levels of metabolites. A metabolite refers to a substance that is produced or consumed during metabolism. Metabolites are intermediates in a metabolic pathway and can be substrates or products. Quantifying metabolites can bring insight into mechanisms by which the environment affects biological/metabolic processes (Walker et al., 2019). For example, a study by Hysi et al. (2019) used this strategy to look at the metabolic associations of intraocular pressure (IOP) which is an endophenotype for glaucoma (Hysi et al., 2019).

Genetic factors can influence the levels of metabolites and there have been recently conducted large-scale genome-wide association studies (GWAS) to identify genetic associations (Lotta et al., 2021; Hysi et al., 2022; Yin et al., 2022; Chen et al., 2023). Chen et al. (2023) recently conducted a GWAS to identify genetic factors associated with all metabolites measured in participants of the Canadian Longitudinal Study on Aging (CLSA) (Raina et al., 2019; Michelotti et al., 2023). Among the metabolites investigated were O-methylascorbate and ascorbic acid 2-sulfate, both of which are involved in vitamin C metabolism (Blaschke and Hertting, 1971; Tolbert et al., 1975). A few single nucleotide polymorphisms (SNPs) were found to be significantly associated with these two metabolites (Chen et al., 2023). Yin et al. (2022), conducted a similar analysis which included both metabolites in a Finnish population and found significant associations (Yin et al., 2022). However, it is not known whether these genetic associations are affected by sex, which may explain some of the sex differences in vitamin C concentrations seen between males and females.

In this paper we aimed to investigate how sex affects the genetic association of variants across the genome with O-methylascorbate and ascorbic acid 2-sulfate in the CLSA data. In addition, using the comprehensive functional annotation platform FUMA (Watanabe et al., 2017), we aimed to uncover the functional consequences of these genetic associations. This would help to investigate potential molecular mechanisms for sex-differences in vitamin C concentrations such as through an association with sex hormones. Understanding how genetic factors affect vitamin C-related metabolites can aid in understanding the influence of vitamin C on metabolic processes, and in turn how vitamin C influences disease phenotypes.

## 2 Methods

### 2.1 Study population

We used baseline data from the Comprehensive Cohort of the Canadian Longitudinal Study on Aging (CLSA) (Raina et al., 2019). The CLSA recruited participants between 2010 and 2015 who were between the ages of 45–85 years to investigate social, environmental, and other factors that affect aging and disease. The Comprehensive Cohort included 30,097 participants with baseline data collected between 2012 and 2015 via in-home interviews and in-person physical examinations and biospecimen sample collections at CLSA data collection sites located in Victoria, Vancouver, Surrey, Calgary, Winnipeg, Hamilton, Ottawa, Montreal, Sherbrooke, Halifax, and St. John’s, Canada. Inclusion criteria required participants to be community dwelling, be cognitively unimpaired, and to speak English or French. Not included were full-time members of the Canadian Armed Forces, those residing on a federal First Nations reserve or settlement, those living in a long-term care institution, and non-residents or citizens of Canada.

Of the Comprehensive Cohort, 9,992 participants had metabolite levels quantified and 26,662 individuals were genotyped (Forgetta et al., 2022; Michelotti et al., 2023). In this study we focused on ∼9,000 CLSA participants of European ancestry with genetic and metabolic information and without any missing covariate information. Written informed consent was obtained for all participants, and research ethics board approval was obtained for all CLSA affiliated sites. The analysis presented here was approved by the University of Ottawa research ethics board.

### 2.2 Genomic data quality control

Blood samples were collected from consenting participants of the CLSA Comprehensive Cohort, and samples were moved to −80°C storage before shipment to the genomics facility where they were stored at −20°C. The Affymetrix Axiom array was used to perform genome-wide genotyping, resulting in 794,409 variants from 26,622 participants (Forgetta et al., 2022). We followed the genetic ancestry procedures performed by the CLSA to identify participants of European descent (Forgetta et al., 2022). The CLSA genomic data release included genotype data imputed using the TOPMed reference panel, resulting in ∼308 million variants imputed (Taliun et al., 2021). Prior to GWAS, we filtered out variants from the imputed data that had a minor allele frequency (MAF) <0.01, an imputation quality score <0.3 and missingness >0.1. After these filters, 8,836,359 variants remained for analysis. Both single nucleotide polymorphisms (SNPs) and insertions/deletions (INDELs) were included. All genomic positions are according to the human reference genome assembly GRCh38/hg38.

### 2.3 Metabolite processing

Metabolite levels were quantified using mass spectrometry and then identified using the Metabolon Discovery HD4TM LC-MS platform (Michelotti et al., 2023). Metabolite values underwent quality control measures and 1,314 metabolites were included in the final dataset. We were interested in two metabolites: O-methylascorbate and ascorbic acid 2-sulfate. In this GWAS we used the CLSA data with batch normalized values and where missing values were imputed with the lowest value recorded. Other researchers have reported using this imputation approach for missing values, and we assumed missing values were due to the limit of detection of the Metabolon platform (Playdon et al., 2019). For O-methyl ascorbate, there were no missing values, and for ascorbic acid 2-sulfate, there were 113 missing values. Prior to analysis, metabolites levels were log-transformed and extreme outliers (more than 3 SD away) removed followed by normalization to a mean of 0 and an SD of one which was done in prior studies (Chen et al., 2023).

### 2.4 Overall genome-wide association study

We first performed a GWAS on the complete dataset using mixed linear models as implemented in the GCTA/fastGWA program (Jiang et al., 2019). Briefly, the model fit by GCTA/fastGWA is:

$$y=x_{snp}\beta_{snp}+X_{c}\beta_{c}+g+e$$

where y is the vector of metabolite levels, $x_{snp}$ is the vector of genotypes of a specific genetic variant, $\beta_{snp}$ is the coefficient of the genetic variant, $X_{c}$ is the matrix of fixed covariates with their respective coefficients $\beta_{c}$ , g captures the total genetic effects with $g\;∼N(0,\pi \sigma_{g}^{2}$ ) and relatedness matrix π , and e is the residual effect with $e\;∼\;N\left(0,I\sigma_{e}^{2}\right)$ . We used a sparse SNP-derived genetic relatedness matrix (GRM) as a covariance structure to control for population stratification and relatedness. The covariates included in the model were age, sex, batch number, the first ten genetic principal components, province, and hours since the last meal or drink.

After removing individuals with missing covariate values and outlier metabolites, there were 8,916 participants for the O-methylascorbate GWAS and 8,835 participants for the ascorbic acid 2-sulfate GWAS. Manhattan plots and qqplots were made using the qqman (Turner, 2018) package in R to visualize for any statistically significant variants. We only followed up genome-wide significant genetic variants (*p*-value <5 × 10^−8^). In our Supplementary Tables SA1, we also provide our results for variants meeting the suggestive level of significance (*p*-value <1 × 10^−5^) (Duggal et al., 2008) for ease of replication in future studies.

Independent (i.e., in linkage equilibrium) genetic variants were obtained using the GCTA/COJO program (Yang et al., 2012) which implements a stepwise selection procedure to identify variants within significantly associated genomic regions that remain independently associated with the trait after conditioning on most statistically significant variants. The program also incorporates linkage disequilibrium (LD) structure information from an input population, which was set as the same individuals as those used in the GWAS analysis. Significant variant threshold was based on the genome-wide significance level of 5 × 10^−8^.

We investigated functional significance using the platform FUMA (Watanabe et al., 2017) for lead variants identified by COJO analysis. To identify associated genes, positional mapping, quantitative expression quantitative trait loci (eQTL) mapping, and chromatin interaction mapping were used. FUMA identifies all variants in LD (based on 1000 Genomes LD structure) with lead variants to use for mapping. Variants were filtered prior to mapping to only those with a Combined Annotation Dependent Depletion (CADD) (Rentzsch et al., 2019) score above a threshold established as associated with variants with deleterious effects (Amendola et al., 2015; Watanabe et al., 2017) that based on research classifying the pathogenicity of genetic variants was set at the suggested level of >12.37 (Amendola et al., 2015). For positional mapping, we additionally only used exonic or splicing variants. For eQTL mapping, a false discovery rate (FDR) threshold of <0.05 was adopted. For chromatin interaction mapping the threshold was an FDR of <1 × 10^−06^, in line with the default FUMA parameters (Watanabe et al., 2017). All tissues were used for mapping.

### 2.5 Genetic variant-sex interaction GWAS

We performed a GWAS to test whether genetic effects were modified by chromosomal sex. To achieve this, we used the GCTA program fastGWA-GE (Zhong et al., 2023), which fits the following model:

$$y=G\beta_{G}+\left(G\circ E\right)\beta_{GEI}+X_{c}\beta_{c}+g_{\rho }+\epsilon$$

where ∘ is the Hadamard element-wise product. In this model, y is the n×1 vector of phenotypes, G is the vector of genotypes of a specific genetic variant, E is the vector of standardized environmental variable (here chromosomal sex) and $\beta_{GEI}$ is the gene-environment interaction (GEI) effect while $X_{c}$ is the matrix of covariates with effects $\beta_{c}$ . The vector of residuals $\epsilon \;∼\;N\left(0,I_{n}\sigma_{\epsilon }^{2}\right)$ where $I_{n}$ is an n×n identity matrix. The vector $g_{\rho }$ is an n×1 vector of all combined genetic main and GEI effects with $g_{\rho }∼\;N\left(0,K_{\rho }\sigma_{g}^{2}\right)$ , where $K_{\rho }=\rho K+\left(1-\rho \right)DKD$ , K is the kinship matrix (or a sparse GRM), D is an n x n matrix where the jth diagonal entry is $E_{j}$ , $\rho =\frac{\sigma_{\text{main}}^{2}}{\sigma_{g}^{2}}$ , and $\sigma_{g}^{2}=\sigma_{\text{main}}^{2}+\sigma_{GEI}^{2}$ . In fastGWA-GE, the variance components $\sigma_{g}^{2}$ and $\sigma_{\epsilon }^{2}$ , and ρ are estimated under the null hypothesis of no genetic or GEI effects. These estimates are then used to obtain a residualized phenotype, $y_{\text{resid}}=y_{\text{adj}}-{\hat{g}}_{\rho }$ , by removing the predicted genetic effects ${\hat{g}}_{\rho }={\hat{\sigma }}_{\epsilon }^{2}V^{-1}y_{\text{adj}}$ , where $y_{\text{adj}}=y-X_{c}{\left(X_{c}^{T}X_{c}\right)}^{-1}X_{c}^{T}y$ and $V^{-1}$ is obtained from the estimates of $\sigma_{g}^{2}$ , $\sigma_{\epsilon }^{2}$ , and ρ . The test of $\beta_{GEI}=0$ after adjusting for genetic main effects is then performed by a Wald test with sandwich correction from the linear regression: $y_{\text{resid}}=G\beta_{G}+\left(G\circ E\right)\beta_{GEI}+\epsilon$ .

We adjusted for the covariates age, batch number, province, hours since last meal and the first 10 principal components. We used the same sparse GRM as for the main GWAS above and filtered for MAF 0.01 using the GCTA program, leading to 8,580,042 variants.

### 2.6 Finding significant signals of interaction and their functional consequences

We followed up all suggestive interaction signals based on a *p*-value threshold commonly used for suggestive significance in GWASs (*p*-value of interaction test <1 × 10^−05^) (Duggal et al., 2008). We used the FUMA application to select lead variants and define loci of interest. The same parameters were used as before, such as filtering by CADD score; however, lead variants were not provided to FUMA externally. In this case, the application selects lead variants and independent significant variants based on LD (*r* ^2^) information (Watanabe et al., 2017). In brief, all variants with *p*-values below the suggestive threshold and independent from each other (*r* ^2^ < 0.6) were identified. Variants that were in *r* ^2^ ≥ 0.6 with these variants and had a *p*-value less than 0.05 were equally considered for gene mapping. Lead variants were chosen from the identified suggestive variants if they had *r* ^2^ < 0.1 with other shortlisted variants.

In a small number of cases (8), variants that were suggestive were not recognized in the FUMA application, and required an alternative investigation. These variant regions were visualized using LD Link (Machiela and Chanock, 2015) and lead variants and any additional annotated variants were selected based on similar criteria to FUMA. The CADD score of resulting variants was determined to filter variants that could be used for mapping. None of the identified variants were above the CADD threshold for deleteriousness and therefore were not mapped to genes. To annotate the functions of the lead variants and variants that met FUMA criteria, but which were not recognized in the platform, the Variant Effect Predictor (VEP) platform from Ensembl was used (McLaren et al., 2016).

To follow-up on interaction results, we estimated the effect sizes for each lead variant in linear mixed models after stratifying the sample by sex. The models were the same as for the overall GWAS described above and were adjusted for the same covariates. For O-methylascorbate, the number of participants was 4,580 and 4,329 for males and females, respectively. For ascorbic acid 2-sulfate, the number of participants was 4,310 and 4,518 for males and females, respectively.

To illustrate the direction of effect for each lead identified variant stratified by sex, we plotted interaction graphs using the results from the GWAS stratified by sex. We plotted the effect size of each lead variant by sex with a 95% confidence interval using R/ggplot.

To report the results of this study, we were informed by Strengthening the Reporting of Genetic Associations (STREGA) guidelines (Little et al., 2009).

## 3 Results

### 3.1 Overall GWAS results

Using the GCTA-fastGWA program we found 592 statistically significantly associated variants (*p*-value <5 × 10^−8^) with O-methylascorbate. For ascorbic acid 2-sulfate, we found 56 statistically significantly associated variants (*p*-value < 5 × 10^−8^). The Manhattan plot of these results is displayed in Supplementary Figure S1 and Supplementary Figure S2. The full list of suggestive variants and *p*-values for O-methylascorbate and ascorbic acid 2-sulfate are found in Supplementary Table S1 and Supplementary Table S2 respectively.

Using the GCTA-COJO software to follow-up significantly associated variants (*p*-value < 5 × 10^−8^), we identified three independent lead variants for O-methylascorbate, all of which were on chromosome 22. For ascorbic acid 2-sulfate, we identified two lead variants on chromosome 16 and one lead variant at chromosome 10. The variant positions, effect allele frequencies, GWAS *p*-value and COJO-adjusted *p*-value are summarized in Table 1.

**TABLE 1:** Lead variants from overall GWAS analysis selected using GCTA-COJO. Details of chromosome, position, effect allele frequency, *p*-value from GWAS, and COJO-adjusted *p*-value. Variants were selected using the GCTA-COJO program.

O-methylascorbate				
Chr	Variant	Effect Allele Frequency	*p*-value	COJO-adjusted *p*-value
22	chr22:19953481:A:G	0.95	4.62 × 10^−22^	8.24 × 10^−09^
22	chr22:19959676:CTCT:C	0.15	4.35 × 10^−11^	1.13 × 10^−29^
22	chr22:19963748:G:A	0.52	5.20 × 10^−214^	3.24 × 10^−204^

Ascorbic Acid 2-sulfate				
Chr	Variant	Effect Allele Frequency	*p*-value	COJO-adjusted *p*-value
10	chr10:87718088:G:A	0.38	3.42 × 10^−08^	3.61 × 10^−08^
16	chr16:30343759:C:T	0.02	1.31 × 10^−12^	2.94 × 10^−11^
16	chr16:30374516:T:C	0.73	2.12 × 10^−11^	4.70 × 10^−10^

### 3.2 Functional consequences of genes

Using the FUMA platform, we determined the functional consequences of the lead variants found in the COJO analysis and variants in LD with those variants which met FUMA criteria. Variants associated with O-methylascorbate were mapped to 13 genes, which included both known and novel gene associations (Supplementary Table S5). We subsequently used the FUMA platform to identify the functional consequences of the mapped genes, such as their expression levels in different tissue types and whether the set of genes was enriched in any functional pathways. The mapped genes were not significantly differentially expressed in any tissue types (Supplementary Table S6). The functional gene sets linked to some processes such as genes which are involved in a cancer cell-death evasion mechanism (Jinesh and Kamat, 2017). No gene sets could be directly related to vitamin C functions (Supplementary Table S7).

Variants associated with ascorbic acid 2-sulfate were mapped to 71 genes (Supplementary Table S8). Using the same process as before, we found that the mapped genes were not significantly differentially expressed in any tissue types (Supplementary Table S9). The mapped genes were linked to gene sets for chromosomal and proximal deletions syndromes; however, they did not link to any vitamin C functions (Supplementary Table S10). Several novel genes were mapped to ascorbic acid 2-sulfate as well.

### 3.3 Genetic variant-sex interaction GWAS

**FIGURE 1:**
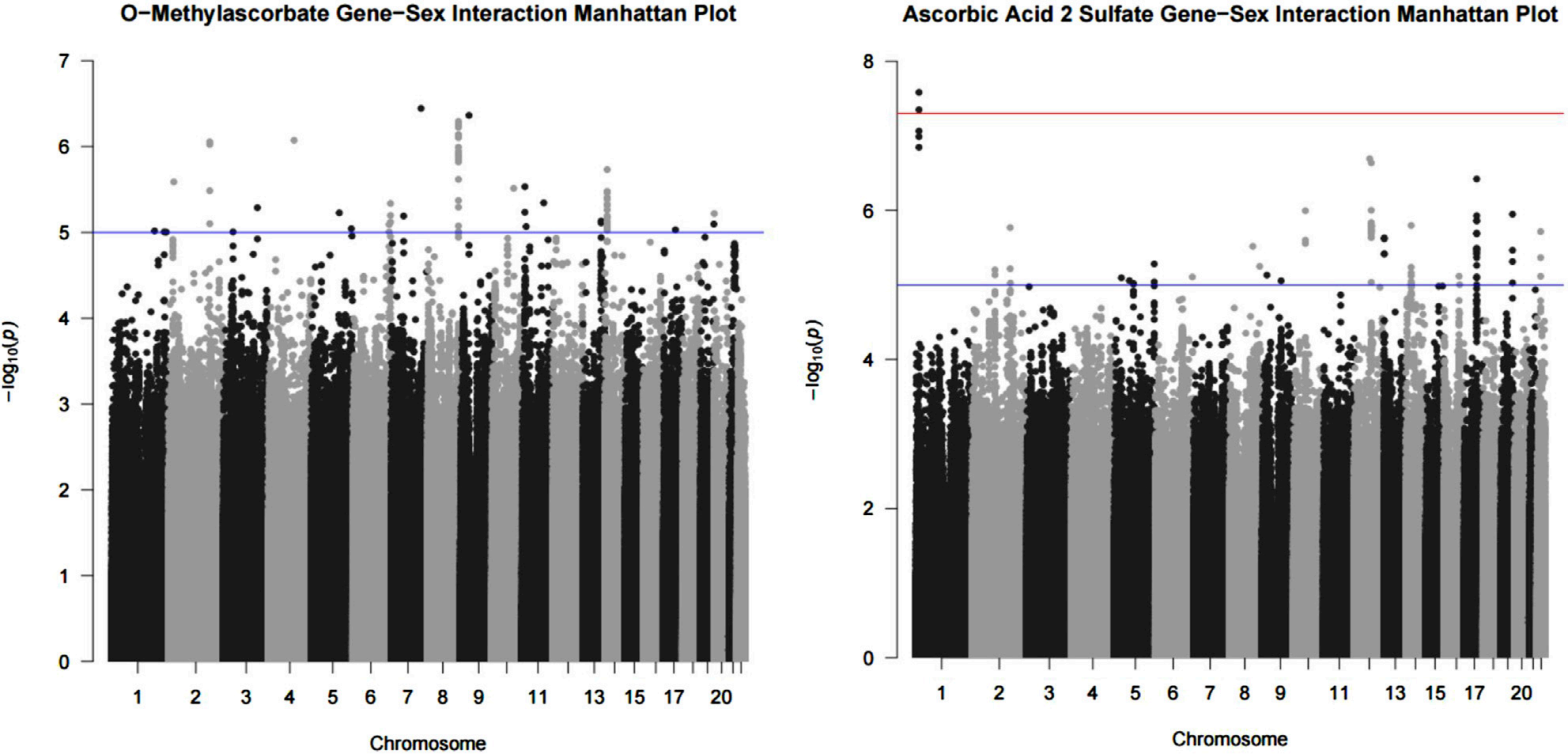
O-methylascorbate and ascorbic acid 2-sulfate interaction with sex results. Results from the GWAS analysis using the FastGWA-GE program from GCTA in the form of a Manhattan plot. GWAS conducted using a mixed linear model adjusted for age, batch number, province, 10 principal components, and hours since last meal or drink which incorporated a genetic relatedness matrix to account for population stratification. The models also included an interaction term for sex. Each point represents the *p*-value of the interaction between sex and the variant on the associated chromosome. The red line is the significant (5 × 10^−8^) threshold and the blue line is the suggestive (1 × 10^−5^) threshold.

We conducted a genetic variant-by-sex GWAS and found that, for O-methylascorbate, there were no statistically significant interactions while there were 69 suggestive interaction variants. For ascorbic acid 2-sulfate, we found two statistically significant interaction variants and 83 suggestive interaction variants. The significant variants were rs1296721356 (chr1:13354706) and rs1301173408 (chr1:13341668). Manhattan plots visualizing the associations are shown in Figure 1. The full list of suggestive interaction variants and *p*-values for O-methylascorbate and ascorbic acid 2-sulfate are found in Supplementary Table S3 and Supplementary Table S4 respectively.

**FIGURE 2:**
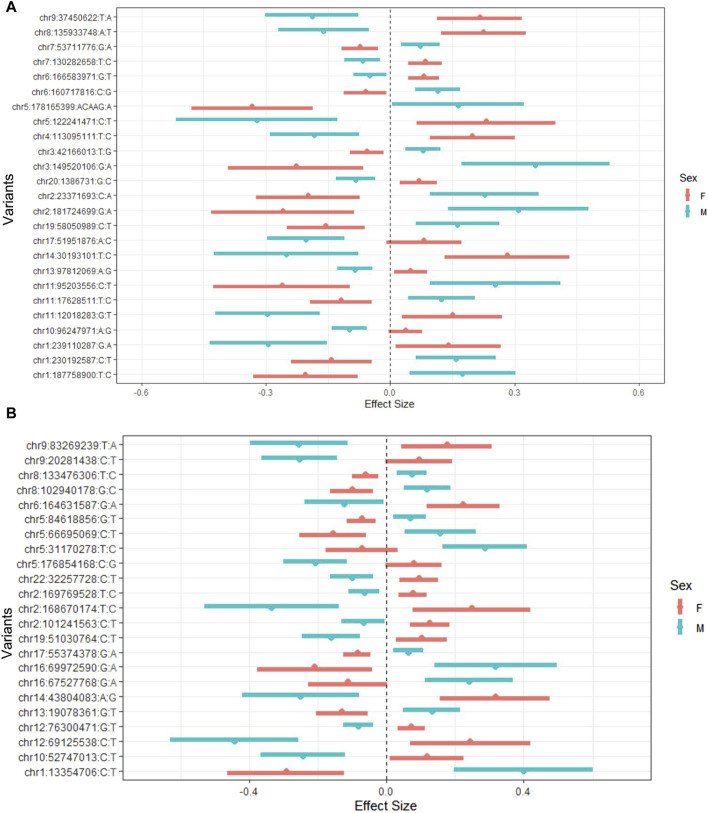
O-methylascorbate **(A)** and ascorbic acid 2-sulfate **(B)** effect sizes of lead interacting variant by sex. Effect sizes and 95% confidence intervals from the GWAS stratified by sex. GWAS conducted using a mixed linear model adjusted for age, batch number, province, 10 principal components, and hours since last meal or drink which incorporated a genetic relatedness matrix to account for population stratification. The list of variants are the lead variants for each suggestive locus found in the interaction GWAS analysis. Red represents female values and blue represents male values.

We investigated all the signals at statistically significant and suggestive levels. Using the default parameters of FUMA, we identified 25 lead variants for O-methylascorbate and 23 lead variants for ascorbic acid 2-sulfate (Supplementary Tables S11, S12). After conducting separate sex stratified GWAS analyses for comparison, we saw that all interactions were qualitative in nature, meaning that the variants had opposite effects in males and females in the sex-stratified analyses (Figure 2).

In Tables 2, 3, we summarized the lead variants for each suggestive interaction loci, nearest genes, annotated functions, and number of genes mapped at each suggestive genomic loci for O-methylascorbate and ascorbic acid 2-sulfate respectively. Overall, most of the genomic loci included variants in intronic or intergenic regions, which were not mapped to any genes. However, some regions had variants of likely functional importance which were mapped to more than one gene.

**TABLE 2:** O-methylascorbate suggestive interaction loci functions. Lead variants for each suggestive interaction locus were selected based on FUMA criteria. For each locus, variants in LD of the lead variant were analyzed to note down the nearest gene and function. The number of genes mapped to each locus is based on FUMA criteria. Loci that did not register in FUMA, and whose functions and nearest gene information were retrieved using VEP are marked.

Locus	Locus lead variant RSID	Locus lead variant position	Nearest gene	Function	# Genes mapped	Interaction *p*-value
1	rs561458908	chr1:187758900:T:C	*RP5-925F19.1*	Intergenic	0	9.61 × 10^−6^
2	rs6673444	chr1:230192587:C:T	*GALNT2*	Intronic/Exonic	0	9.86 × 10^−6^
3	rs74527587	chr1:239110287:G:A	*RP11-307O1.1*	Intergenic	0	9.91 × 10^−6^
4	rs13427429	chr2:23371693:C:A	*AC012506.4*	Intergenic	0	2.58 × 10^−6^
5	rs183979544	chr2:181724699:G:A	*CERKL/AC013733.5/RUN6ATAC19)/PDE1A*	Intronic/UTR5/Intergenic	0	8.81 × 10^−7^
6	rs6775014	chr3:42166013:T:G	*TRAK1*	Intronic	0	9.86 × 10^−6^
7	rs139707525	chr3:149520106:G:A	*WWTR1*	UTR3	0	5.15 × 10^−6^
8	rs74597555	chr4:113095111:T:C	*ANK2*	Intronic	0	8.44 × 10^−7^
9	rs2656901	chr5:175120419:G:A	*CTC-281M20.1/ARL2BPP6*	Intergenic	0	9.06 × 10^−6^
10	rs783146	chr6:160717816:C:G	*PLG*	Intronic/Intergenic	0	8.10 × 10^−6^
11	rs3823198	chr6:166583971:G:T	*RPS6KA2*	Intronic	0	4.60 × 10^−6^
12	rs11764886	chr7:53711776:G:A	*GS1-179L18.1*	Intergnic/ncRNA_intronic/	3	6.43 × 10^−6^
13	rs6952090	chr7:130282658:T:C	*RP11-190G13.4/CPA2*	NcRNA_intronic/Intergenic/Downstream/Intronic/Upstream	13	3.59 × 10^−7^
14	rs2922384	chr8:135933748:A:T	Several	NcRNA_intronic/Intergenic/NcRNA_exonic/Intronic/Upstream	2	5.08 × 10^−7^
15	rs116934390	chr9:37450622:T:A	*ZBTB5*	Intronic	0	4.33 × 10^−7^
16	rs10882762	chr10:96247971:A:G	*BLNK*	Intronic	0	3.07 × 10^−6^
17	rs79414703	chr11:12018283:G:T	*RP13-631K18.2/DKK3*	Intergenic/Intronic	0	2.93 × 10^−6^
18	rs79157408	chr11:17628511:T:C	*OTOG*	Intronic	0	8.56 × 10^−6^
19	rs80136724	chr11:95203556:C:T	*RP11-712B9.2:SESN3*	NcRNA_Intronic	0	4.53 × 10^−6^
20	rs7331036	chr13:9,7812069:A:G	*snoU13*	Intergenic	1	7.38 × 10^−6^
21	rs75638828	chr14:30193101:T:C	*PRKD1:CTD-2251F13.1*	NcRNA_intronic	2	1.85 × 10^−6^
22	rs74665914	chr17:51951876:A:C	*CA10*	Intronic	2	9.33 × 10^−6^
23	rs76958646	chr19:58050989:C:T	*ZNF135/ZSCAN1*	Intronic	0	8.00 × 10^−6^
24	rs6041909	chr20:1386731:G:C	*FKBP1A*	Intronic	0	6.03 × 10^−6^
25^a^	rs1470819913	chr5:122241471:C:T	*ZNF474*	Intergenic	0	5.92 × 10^−6^

^a^ Features annotated using VEP, platform, genetic region not available in FUMA.

**TABLE 3:** Ascorbic acid 2-sulfate suggestive interaction loci functions. Lead variants for each suggestive interaction locus were selected based on FUMA criteria. For each locus, variants in LD of the lead variant here analyzed to note down the nearest gene and function. The number of genes mapped to each locus is based on FUMA criteria. Loci that did not register in FUMA, and whose functions and nearest gene information were retrieved using VEP are marked.

Locus	Locus lead variant RSID	Locus lead variant position	Nearest gene	Function	# Genes mapped	Interaction *p*-value
1	rs1192803	chr2:101241563:C:T	Several	Intergenic/Intronic/Downstream/Exonic	0	6.29 × 10^−6^
2	rs142867333	chr2:168670174:T:C	*CERS6*	Intronic	0	1.70 × 10^−6^
3	rs12692923	chr2:169769528:T:C	*KLHL23/KLHL23:PTCHD3P2*	Intronic/ncRNA_exonic/UTR3	0	9.45 × 10^−6^
4	rs7720392	chr5:31170278:T:C	*RP11-152K4.2*	NcRNA_intronic	1	8.03 × 10^−6^
5	rs114959781	chr5:66695069:C:T	*MAST4*	Intronic	0	8.76 × 10^−6^
6	rs28579435	chr5:84618856:G:T	*CTD-2269F5.1*	Intergenic	3	9.58 × 10^−6^
7	rs4976655	chr5:176854168:C:G	*UNC5A/HK3*	Intronic/UTR3	3	5.23 × 10^−6^
8	rs13204324	chr6:164631587:G:A	*RP11-300M24.1*	Intergenic	0	7.83 × 10^−6^
9	rs62526542	chr8:102940178:G:C	*AZIN1:KB-1507C5.2/KB-1507C5.2*	Intronic/Intergenic/Upstream	0	3.03 × 10^−6^
10	rs1048471	chr8:133476306:T:C	*ST3GAL1*	Intronic/Exonic/UTR5	0	5.64 × 10^−6^
11	rs11792574	chr9:20281438:C:T	*AL512635.1*	Intergenic	0	7.38 × 10^−6^
12	rs117196678	chr9:83269239:T:A	*RP11-439K3.1/FRMD3*	NcRNA_intronic/Intronic	0	8.81 × 10^−6^
13	rs117271698	chr10:52697952:C:T	*RP11-556E13.1/MBL2*	ncRNA_intronic/Intergenic	0	1.01 × 10^−6^
14	rs117414281	chr12:69125538:C:T	*AC139931.1*	Intergenic	0	2.03 × 10^−7^
15	rs10859572	chr12:76300471:G:T	*RP11-54A9.1*	ncRNA_intronic/ncRNA_exonic	0	2.31 × 10^−7^
16	rs10162161	chr13:19078361:G:T	*RNA5SP24*	Intergenic/Downstream	0	2.34 × 10^−6^
17	rs76250049	chr14:43804083:A:G	*RP11-305B6.3*	Intergenic	0	1.59 × 10^−6^
18	rs35031569	chr16:67527768:G:A	Several	Exonic/Intronic/Upstream/Downstream/Intergenic/UTR3/ncRNA_exonic/ncRNA_Intronic	49	7.65 × 10^−6^
19	rs148001569	chr16:69972590:G:A	*NFAT5/PDXDC2P/ST3GAL2*	Intronic/Intergenic	0	9.92 × 10^−6^
20	rs4334353	chr17:55374378:G:A	*RP11-515O17.3/MMD*	Intergenic	3	3.79 × 10^−7^
21	rs75565227	chr19:51030764:C:T	*KLK/CTC-518B2.10*	NcRNA_Intronic/Upstream/Intergenic	0	1.13 × 10^−6^
22	rs9609471	chr22:32257728:C:T	*RFPL2/RP1-90G24.10:SLC5A4*	Intergenic/Downstream/Intronic/ncRNA_intronic	0	1.93 × 10^−6^
23^a^	rs1296721356	chr1:13354706:C:T	Several	Upstream/Downstream/Intron/Intergenic/UTR	0	2.61 × 10^−8^

^a^ Features annotated using VEP, platform, genetic region not available in FUMA.

Variants rs1470819913 and rs1296721356 were not available in FUMA and were annotated separately. No genes were mapped to these regions, and the summary of their functions is included in Tables 2, 3.

### 3.4 Functional consequences of interaction signals

The variants that interacting with sex were significantly associated with O-methylascorbate levels, were mapped to 23 different genes (Supplementary Table S13). Looking at tissue expression data, the set of genes is statistically significantly differentially expressed (corrected *p*-value <0.05) in testis (Supplementary Table S14).

The variants that interacted with sex for ascorbic acid 2-sulfate were mapped to 59 genes (Supplementary Table S15). Looking at tissue expression data, the set of genes is statistically significantly differentially expressed (correct *p*-value <0.05) in the stomach (Supplementary Table S16). One of the mapped genes was *AGRP* which is a neuropeptide which controls feeding behavior as a stimulating hormone antagonist (Deem et al., 2022). Another gene mapped was *HSD11B2*, which is an enzyme involved in cortisol metabolism, and which has been shown to be controlled by sex hormones (Garbrecht et al., 2007). The significant genomic loci identified on chromosome one was not mapped to any genes and we did not find evidence of a potential functional consequence of this region based on our criteria. For both O-methylascorbate and ascorbic acid 2-sulfate, no gene-sets related to vitamin C metabolism or sex hormones were identified (Supplementary TableS S17, S18).

## 4 Discussion

In the present study, we investigated associations and the functional consequences of genetic variant-metabolite associations for two specific metabolites related to vitamin C: O-methylascorbate and ascorbic acid 2-sulfate. In addition, our study is the first to investigate potential genetic variant-sex interactions influencing these two metabolites by conducting a gene by sex GWAS. We also examined the functionality of these variants of interest and found some associations with hormone-related genes.

Past research has studied the genomic associations of these metabolite levels, and Chen et al., 2023, conducted their study using the CLSA metabolite and genomic data. We aimed to have a more focused analysis with a less stringent threshold to gain a deeper understanding of the two vitamin C-related metabolites specifically. We report the same associations, with rs144009214 for ascorbic acid 2-sulfate and rs61484427 and rs4680 for O-methylascorbate (Chen et al., 2023). We also examined additional lead variants than was previously reported due to our less stringent significance threshold of 5 × 10^−8^ (not corrected for examining all measured metabolites). Chen et al. also identified *COMT* as the closest protein coding genes to the O-methylascorbate variants, which we also identified using FUMA. Using this platform, we were able to find several novel genes that were mapped to our GWAS results and should be investigated more thoroughly. Yin et al., 2022 also identified variant rs4680 as significant with O-methylascorbate in their analysis (Yin et al., 2022).

Chen et al., identified *CD2BP2* as the closest protein coding genes to the ascorbic acid-2 sulfate associated variant, which we also identified in FUMA, among several other genes (Chen et al., 2023; Schlosser et al., 2023 conducted a GWAS on plasma and urine metabolite levels with individuals with chronic kidney disease. In their analysis, ascorbic acid-2 sulfate was associated with rs111894927 which is in the genetic region of the *MAPK3* gene, which we also identified in FUMA (Schlosser et al., 2023). Yin et al. found some genetic associations with ascorbic acid-2 sulfate, however none of their identified putative causal genes were found in our analysis (Yin et al., 2022).

The FUMA analysis from the initial GWAS identified several new genes that were associated with metabolite levels. However, this comprehensive evaluation did not provide any new evidence of mechanisms or pathways through which these gene associations influence metabolite levels. In addition, none of the novel genes associated could be connected to vitamin C metabolism.

Of the mapped genes, *COMT* is a known factor in vitamin C metabolism. O-methylascorbate is a product of O-methylation by the *COMT* gene (Krumsiek et al., 2012). Therefore, this work strengthens an existing connection and further emphasizes the potential importance of this gene in vitamin C metabolism.

Our analysis of a potential sex effect on the gene associations seen for each metabolite found two significant interactions and several suggestive interactions. The effect of sex is an important factor to consider in disease etiology, and in this case, the sex differences of vitamin C concentrations between males and females and not completely understood. One of the proposed theories for the sex differences seen between vitamin C concentrations in males and females is due to influence by sex hormones (Travica et al., 2020). We explored all suggestive interactions to determine their functional significance as well as if there are any connections to genes related to hormone signaling or sex hormones.

For both ascorbic acid 2-sulfate and O-methylascorbate, there were no gene-sets which are enriched and may be related to vitamin C metabolism or sex hormones. Interestingly, the set of genes mapped for ascorbic acid 2-sulfate was significantly differentially expressed in stomach tissues while the set of genes mapped for O-methylascorbate was differentially expressed in testis tissues.

The variants that interacted with sex for ascorbic acid 2-sulfate were mapped to several genes, one of which was *AGRP*, which produces a peptide agonist molecule important for initiating hunger cues. In general, the *AGRP* neurons signal for increased food intake (Deem et al., 2022). In one animal study using a bird model, researchers found that this protein was differentially expressed between male and female chickens (Caughey et al., 2018). Since vitamin C intake is heavily influenced by dietary exposures, this association with a hunger-driving signal protein may be something of further consideration.

Other variants that interacted with sex for ascorbic acid 2-sulfate were mapped to *HSD11B2*. The role of 11-β hydroxysteroid dehydrogenase type 2, the enzyme encoded by the *HSD11B2* gene, is to oxidize cortisol, a glucocorticoid, into its inactive version cortisone (Chapman et al., 2013). Several animal and *in vivo* studies have shown that the activity of this enzyme is regulated by various sex hormones (Darnel et al., 1999; Garbrecht et al., 2007; Wang et al., 2009). In addition, cortisol may potentially affect vitamin C concentrations in the body; however, it is unclear whether this acts in a sex dependent manner (Travica et al., 2020). Overall, this may point to an important mechanism for future research.

This study has several strengths, including the use of high quality genetic and metabolic datasets. In addition, the use of such a comprehensive tool to annotate variant functions allowed us to use several bioinformatics tools to identify novel associations and areas for future research.

This study also had some limitations to consider. One limitation is the sample size, which may have hindered the ability to find more statistically significant associations, especially in the interaction analysis. However, these findings showed several associations at the suggestive level, which could be followed up by other researchers. Future studies should replicate these findings ideally with a larger sample size. Another limitation is that this study was only conducted using participants of European descent to avoid problems with population stratification. The CLSA had a very high percentage of participants of European descent so using populations from other ancestries would not provide enough power for an accurate analysis in those groups. Finally, because we decided to use the suggestive threshold for the interaction signals to follow up with for functional annotation and mapping, there is a possibility that some of these associations represent type I error. As this represents the first analysis of a potential sex interaction, future studies should evaluate these regions with larger sample sizes to determine if they are true associations.

In conclusion, our study found potential evidence for an effect modification by sex of genetic associations with two vitamin C related metabolites. In addition, a comprehensive analysis of the functions of genomic regions showing suggestive evidence of genetic variant-sex interactions led to some insight into potential mechanisms for these differences. Future studies are needed to expand on this analysis and further understand the different mechanisms which influence vitamin C concentrations in the body. Mechanisms influencing vitamin C concentrations have several implications for different disease phenotypes (Age-Related Eye Disease Study Research Group, 2001; Villagran et al., 2021) and are an especially important consideration for older adults.

## Data Availability

The data analyzed in this study is subject to the following licenses/restrictions: Data are available from the Canadian Longitudinal Study on Aging for researchers who meet the criteria for access to de-identified CLSA data. Requests to access these datasets should be directed to www.clsa-elcv.ca. GWAS data from the genetic variant-sex GWAS are deposited in the GWAS catalogue (https://www.ebi.ac.uk/gwas/home), accession numbers GCST90399837 and GCST90399838. Code is made available on GitHub (https://github.com/Roy-Gagnon-lab).
