# Supplementary material for: Effect modification by sex of genetic associations of vitamin C related metabolites in the Canadian Longitudinal study on aging
Source: Front Genet. 2024 Jul 31;15:1411931. doi: 10.3389/fgene.2024.1411931 (PMC11322087; doi:10.3389/fgene.2024.1411931)
Supplement: Supplementary file 1 [file Table1.DOCX]

Supplementary Material

# Supplementary Figures


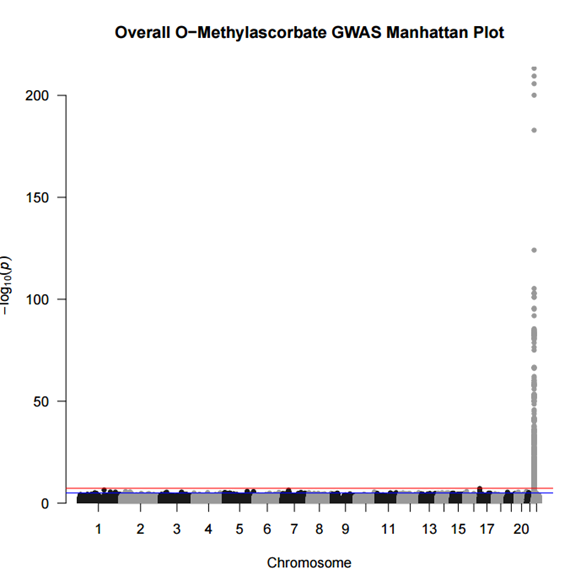


**Figure S1.** **O-methylascorbate** overall **results**. Results from the GWAS analysis using the FastGWA program from GCTA in the form of a Manhattan plot. GWAS conducted using a mixed linear model adjusted for age, sex, batch number, province, 10 principal components, and hours since last meal or drink which incorporated a genetic relatedness matrix to account for population stratification. Each point represents the p-value of a variant on the associated chromosome. The red line is the significant (5 x 10^-8^) threshold and the blue line is the suggestive (1 x 10^-5^) threshold.


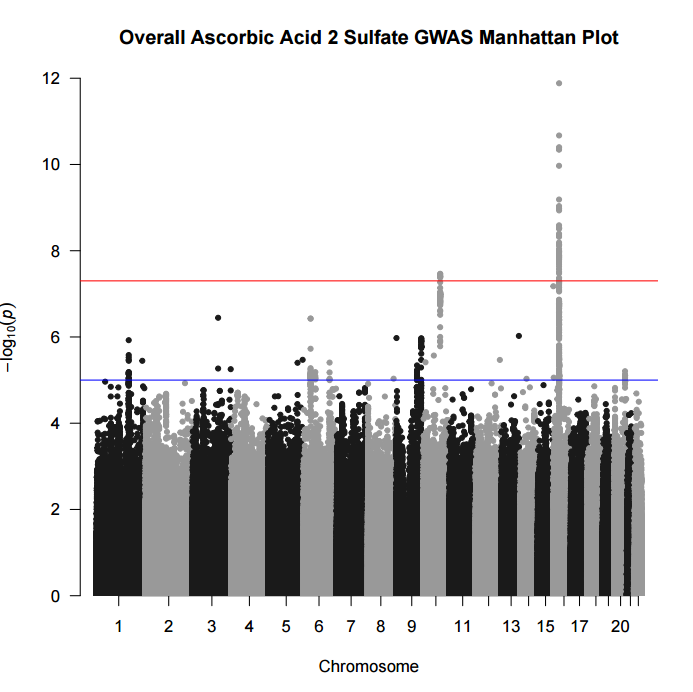


**Figure S2.** **Ascorbic acid 2-sulfate** overall **results**. Results from the GWAS analysis using the FastGWA program from GCTA in the form of a Manhattan plot. GWAS conducted using a mixed linear model adjusted for age, sex, batch number, province, 10 principal components, and hours since last meal or drink which incorporated a genetic relatedness matrix to account for population stratification. Each point represents the p-value of a variant on the associated chromosome. The red line is the significant (5 x 10^-8^) threshold and the blue line is the suggestive (1 x 10^-5^) threshold.
